# Screening for volatile sulphur compounds in a fatal accident case

**DOI:** 10.1080/20961790.2017.1323570

**Published:** 2017-06-07

**Authors:** Ping Xiang, Huosheng Qiang, Baohua Shen, Min Shen

**Affiliations:** a Department of Forensic Toxicology, Shanghai Key Laboratory of Forensic Medicine, Shanghai Forensic Service Platform, Institute of Forensic Science, Ministry of Justice, PRC, Shanghai, China; b Pharmacy School of Wenzhou Medical University, Wenzhou, China

**Keywords:** Forensic science, forensic toxicology, volatile sulphur compounds, HS-GC/FID, screening, fatal accident case

## Abstract

Acute fatal poisoning due to the inhalation of toxic gas frequently occurs in China. Volatile sulphur compounds (VSCs) are toxic to humans. In fatal poisoning investigations, such as those in industrial settings, a number of VSCs, including methanethiol (MT), dimethyl sulphide (DMS), dimethyl disulphide (DMDS) and dimethyl trisulphide (DMTS), can coexist. To date, there is limited data regarding these compounds in post-mortem cases. In the present study, we report toxicological findings in a fatal accident case with two victims. Headspace gas chromatography/flame ionization detector with two columns of different polarities was utilized to screen MT, DMS, DMDS and DMTS in blood. The limits of detection in both methods were 0.05 mg/mL. No sulphur compounds were detected in the blood samples of the two victims. DMS and DMDS were detected in the lungs at concentrations of 0.5 and 1.3 mg/g and 2.2 and 4.1 mg/g, respectively. DMDS liver concentrations were 2.5 and 6.5 mg/g. In addition to hydrogen sulphide, screening for additional VSCs could help establish the cause of death.

## Introduction

Acute fatal poisoning due to the inhalation of toxic gas frequently occurs in China [1,2]. In addition, more than one victim is typically involved [3,4]. In most of these cases, poisoning resulted from accidents in culverts, inspection wells, sewer systems, chemical factories, etc. In addition to carbon monoxide and cyanide, volatile sulphur compounds (VSCs) are always investigated.

VSCs are toxic to humans. The toxic potential of sulphides is derived from the fact that they inhibit the cytochrome oxidase enzyme at the end of the respiratory chain in all aerobic cell mitochondria [5]. In addition to hydrogen sulphide (H_2_S), these gases may also contain methanethiol (methylmercaptan, CH_3_SH, MT), dimethyl sulphide (CH_3_SCH_3_, DMS), dimethyl disulphide (CH_3_SSCH_3_, DMDS) and/or dimethyl trisulphide (DMTS) [6]. These compounds have a bad reputation, mainly because of their toxicity and bad odour. Hydrogen sulphide is extremely toxic with a characteristic “rotten egg” scent. MT and DMS smell similar to rotten cabbage, while DMDS smells like garlic [6].

VSCs can be produced and released from pulp and paper mills and from wastewater treatment plants. The components of VSCs differ when produced from different sources [7]. In fatal poisoning investigations, such as those in industrial settings, H_2_S is a major compound of interest. However, in some accident scenes, considerable concentrations of MT, DMS and DMDS can be found [8]. At high concentration, these compounds are highly toxic and affect the central nervous system.

Sulphide in biological matrices can be measured by pentafluorobenzyl bromide (PFBBr) derivatization using gas chromatography-mass spectrometry (GC-MS) analysis [9]. For highly volatile VSCs, headspace-GC (HS-GC) is the screening method of choice. Several methods have been used to measure DMS and DMDS concentrations in cheese [10], wine [11], seawater [12] and sediments [13]. To date, there is limited data regarding these compounds in post-mortem cases.

This report presents a simple headspace gas chromatography/flame ionization detector (HS-GC/FID) screening method coupled with a procedure for the dual-column confirmation of MT, DMS, DMDS and DMTS in blood. The developed method was applied to determine VSC concentrations in biological samples from two victims in a fatal accident case.

## Case report

At a bamboo pulp mill, two workers (V#1 and V#2) were found dead close to a drain tank. Since other employees reported a “bad odour”, gas poisoning was suspected. The autopsies were conducted approximately two months after death and revealed putrefactive changes. Heart blood, liver and lung samples were extracted for toxicological analysis. Blood samples gave negative results for carbon monoxide, cyanide, ethanol and drugs.

## Experimental

### Materials and reagents

Sodium MT (reagent grade, 20% in water) and DMS (reagent grade, >99.7%) were purchased from Shanghai Macklin Biochemical Co., Ltd. (Shanghai, China). DMDS (reagent grade, ≥98%) was obtained from TCI Shanghai Chemical Industry Co., Ltd. (Shanghai, China). DMTS (reagent grade, ≥98%) was obtained from J&K Scientific, Ltd. (Shanghai, China). The internal standard (IS) methylbenzene (analytical grade, ≥99%) was purchased from the Shanghai Chemical Reagent Corporation (Shanghai, China). Deionized water was purified using a Milli-Q system (Millipore, MA, USA). Drug-free human whole blood samples were supplied by the Shanghai Blood Center (Shanghai, China).

### Preparation of calibration, control and IS solutions

A 0.01 mg/mL methylbenzene solution of water was used as the IS. All of the compounds, MT, DMS, DMDS and DMTS, had low water solubility. Because MT is easily oxidized, a single solution of MT and a mixture of DMS, DMDS and DMTS with concentrations of 0.8 mg/mL were prepared in water immediately prior to use. Working solutions for calibration were prepared by dilution of the stock solutions in water. The concentrations of the calibration were 0.1, 0.2, 0.4, 0.5, 0.6 and 0.8 mg/mL, respectively.

First, 80 mg of DMS, DMDS and DMTS were weighed in a 100 mL volumetric flask, diluted with water to the mark and mixed well. Then, 1.250, 3.125 and 8.750 mL of this solution was added to 10 mL volumetric flasks and diluted with water to the mark to obtain control samples with concentrations of 0.1, 0.25 and 0.7 mg/mL, respectively.

### Instrumentation

The chromatographic system used was an HS-GC/FID Agilent 7890A equipped with a FID and coupled to an Agilent 7697A HS sampler. A dual-column confirmation, which involves injecting a single sample and running it through two chromatographic columns, was performed. The two columns had different polarities and consisted of an Agilent DB-ALC1 column with dimensions of 30 m × 0.32 mm, 1.8 μm and an Agilent DB-ALC2 column with dimensions of 30 m × 0.32 mm, 1.2 μm.

The chromatographic gradient was programmed as follows: an initial oven temperature of 40 °C was held for 3 min and then increased in a linear fashion to 150 °C at 10 °C/min and held for 15 min. At the end of each run, the initial temperature was reset to the initial condition and held for 2 min. The injection port was maintained at 150 °C and had a split ratio of 10:1. The detectors were held at 300 °C. The gas flow rates were as follows: hydrogen 30.0 mL/min, air 400.0 mL/min and nitrogen 25.0 mL/min. The nitrogen flow rate was maintained at a constant 17 psi.

The HS injection port was maintained at 65 °C, the loop was maintained at 105 °C and the transfer line was maintained at 110 °C. Before injection of the sample, the vials were incubated for 10 min at 65 °C. The injection time was held constant at 1 min. The GC run time was 24 min. The analytical data were processed using the Agilent ChemStation Rev. B.04.03 software.

### Sample preparation

For preparation, 0.1 mL of blood or 0.1 g of minced tissue was diluted with 0.5 mL of the IS solution in a 10 mL HS vial, which was then immediately sealed with an aluminium cap and silicone polytetrafluoroethylene (PTFE) septum.

### Method validation

The method was validated according to the guidelines for forensic toxicology [14]. The parameters studied were selectivity, limit of detection (LOD), limit of quantification (LOQ), linearity, precision and accuracy.

The selectivity of the method was evaluated by analysing six post-mortem and six clinical blood samples collected from drug-free persons to exclude any interference from endogenous peaks.

The sensitivity of the method was assessed by determining the LOD and LOQ. The calculations of LOD and LOQ were based on signal-to-noise ratios of 3 and 10, respectively. Linearity for the two calibration standards was obtained using six spiked samples having concentrations ranged from 0.1 to 0.8 mg/mL.

The calibration curves (*y* = a*x* + b) were constructed by plotting the observed peak areas for DMS, DMDS and DMTS with respect to the IS versus the analyte concentration (*x*, ng/mL) of the calibration solutions.

The accuracy and precision were calculated by analysing six independent spiked samples with concentrations of 0.1, 0.25 and 0.7 mg/mL. The precision, expressed as the relative standard deviation (RSD) of the calculated concentrations, should not exceed 15%. The accuracy was expressed as the per cent deviation of the observed mean values from the nominal value and was expected to be less than 15%.

For MT, quantitation was only performed for the biosamples. The LOD was estimated quickly after sample preparation.

### Determination of sulphide levels in the biosamples

According to Kage's method [9], sulphide levels in the biosamples were determined by a previously published GC-MS method [15]. To a mixture of 0.5 mL of 20 mol/L PFBBr solution in toluene, 2.0 mL of 10 mol/L 1,3,5-tribromobenzene (TBB) in ethyl acetate (IS solution) and 0.8 mL of 5 mol/L tetradecyldimethylbenzylammonium chloride solution in oxygen-free water saturated with sodium tetraborate and 0.2 mL of blood or 0.2 g of minced tissue were added. The preparation was vortexed for 1 min, and 0.1 g of potassium dihydrogenphosphate was added to the mixture. After agitation for 10 s and centrifugation for 10 min, the supernatant layer was transferred and 0.2 μL of the solution was injected into the GC-MS apparatus.

GC-MS analysis was conducted using a 7890A gas chromatograph (Agilent Technology Inc., CA, USA) interfaced to a 5975 mass selective detector (Agilent Technology Inc., CA, USA). The analytical column was an Agilent HP5 capillary column (30 m × 0.25 mm, 0.25 μm). The carrier gas (He) flow was constant at 1 mL/min. The oven temperature programme was as follows: initially held at 100 °C for 1.5 min, increased to 280 °C at a rate of 40 °C/min and held for 5 min at 280 °C. The mass detector was operated at 70 eV in electron impact ionization mode. Selected ion monitor (SIM) ions for the targets were as follows: *m*/*z* 161, 181 and 394 for bis(pentafluorobenzyl)sulphide (C_6_F_5_CH_2_SCH_2_C_6_F_5_) [retention time (RT) = 5.4 min] and *m*/*z* 235 and 314 for TBB (RT = 4.8 min). The method was fully validated. The standard curve for sulphide was obtained by plotting the peak area ratio of the derivative (*m*/*z* 394) of sulphide to the TBB (*m*/*z* 314) versus sulphide concentration. The LOD and LOQ of sulphide were 0.05 and 0.2 μg/mL, respectively.

## Results and discussion

**Figure 1.**
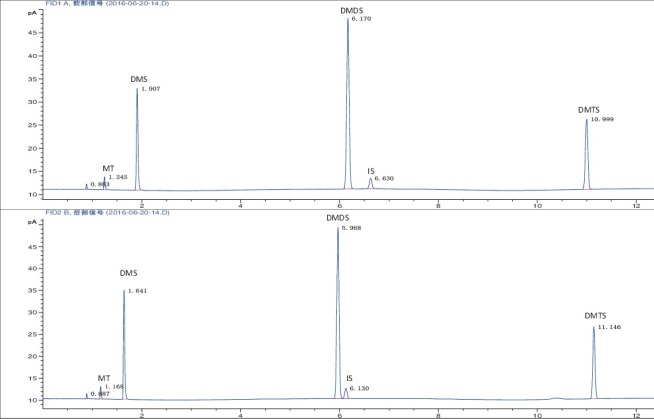
Chromatogram obtained for a mixture of VSCs by HS-GC/FID.

MT, DMS, DMDS and DMTS were well separated on the two different columns, as shown in Figure 1. The IS and all blank samples were free of interfering peaks at the retention times of the target substances.

**Figure 2.**
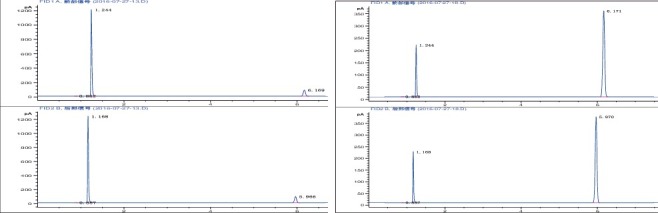
MT oxidization at room temperature (left: 10 min after preparation; right: three days after preparation).

MT is easily oxidized and is a precursor for several other sulphur compounds [16]. After storing a 0.8 mg/mL MT solution at room temperature for three days, more than 80% of the MT oxidized to DMDS (Figure 2). After one week, no MT was detected in the solution. Even when the MT solution was prepared immediately prior to use, DMDS could still be detected. Therefore, method validation was only performed for DMS, DMDS and DMTS.

Calibration curves were produced for each compound over the concentration ranges listed in Table 1. The LOD and LOQ were 0.05 and 0.1 mg/mL, respectively, for DMS, DMDS and DMTS on the two columns. The LOD of MT was approximately 0.05 mg/mL. Data for the accuracy and precision (Table 2) were within the required limits. Inter-day and intra-day precisions were less than 12%.

**Table 1.** Calibration curves for DMS, DMDS and DMTS.

		Column 1		Column 2	
Compound	Calibration ranges (mg/mL)	Calibration curves	*R*^2^	Calibration curves	*R*^2^
DMS	0.1–0.8	*y* = 126.7*x*-9.644	0.998	*y* = 119.7*x*-8.584	0.997
DMDS	0.1–0.8	*y* = 143.6*x*-9.595	0.988	*y* = 135.8*x*-8.406	0.996
DMTS	0.1–0.8	*y* = 23.81*x*-1.179	0.999	*y* = 22.50*x*-0.998	0.998

**Table 2.** Intra-day and inter-day precision and accuracy (*n* = 24, six replicates per day for four days). (%)

		Column 1				Column 2			
Compound	Spiked concentration (mg/mL)	Intra-day precision (RSD, *n* = 6)	Intra-day accuracy (bias, *n* = 6)	Inter-day precision (RSD, *n* = 24)	Inter-day accuracy (bias, *n* = 24)	Intra-day precision (RSD, *n* = 6)	Intra-day accuracy (bias, *n* = 6)	Inter-day precision (RSD, *n* = 24)	Inter-day accuracy (bias, *n* = 24)
	0.1	0.5	−10.3	9.3	−8.1	0.6	−14.3	8.2	−12
DMS	0.25	5.8	−9.7	9.5	−7.7	8.6	−12.8	10.0	−14.4
	0.7	4	−3.3	4.6	−7.8	4.0	−1.2	4.6	−9.8
	0.1	0.4	−10.5	11.1	−8.5	0.6	−12.6	10.9	−12.1
DMDS	0.25	7.8	−6.1	11.9	−10.5	7.7	−7.1	11.9	−14.8
	0.7	9.8	−7.5	8.9	−7.8	9.9	−6.0	6.4	−8.5
	0.1	1.4	−5.5	11.3	−14.1	1.4	−13.9	10.6	−13.0
DMTS	0.25	7	−5.8	10.0	−12.4	7.5	−8.0	8.0	−12.3
	0.7	5.7	−4.7	8.3	−8.3	5.0	−3.9	7.5	−7.7

The calibration concentrations were over a limited range from 0.1 to 0.8 mg/mL because the VSCs are hardly soluble in water. The solubility data for most VSCs in water are in general quite scarce [17]. For example, the water solubility of DMDS is 2.5 g/L (20 °C). The highest concentration of DMS, DMDS and DMTS was 0.8 mg/mL to ensure dissolution completely and stably in water. The method we developed using two columns meets the requirements for the determination of DMS, DMDS and DMTS in post-mortem cases.

The validated method was applied to biological samples obtained from two victims. A tenfold dilution with water was used if the sample concentration exceeded the highest concentration on the calibration curve. The results are shown in Table 3.

**Table 3.** VSCs concentrations in autopsy samples from two victims.

Sample	Sulphide (μg/mL(g))	MT (mg/mL(g))	DMS (mg/mL(g))	DMDS (mg/mL(g))	DMTS (mg/mL(g))
V#1 blood	1.3	−	−^a^	−	−
V#1 lung	1.2	−	−	−	−
V#1 liver	1.7	−	−	−	−
V#2 blood	1.3	−	−	−	−
V#2 lung	1.6	−	−	−	−
V#2 liver	1.6	−	−	−	+^b^

a: −, not detected.

b: +, detected, but below LOQ.

No sulphur compounds of MT, DMS, DMDS and DMTS were detected in the blood samples from V#1 or V#2. DMS and DMDS were detected in the lungs at 0.5 and 1.3 mg/g and 2.2 and 4.1 mg/g, respectively. The DMDS concentration in the liver was 2.2 and 6.5 mg/g. In addition, trace DMTS was found in the liver of V#2.

VSCs are distributed throughout the tissue after inhalation. MT, DMS and DMDS are the most common compounds found in pulp mills [18]. Because of their unique physicochemical properties, nearly all MT exists in the undissociated volatile form [6]. Free MT in aqueous samples (blood and urine) easily volatilizes in open air and escapes detection. We compared this study to Takahama's results [19] of a fatal case of acute MT poisoning. The autopsy of the victim was performed only one day after death. The concentrations of MT in the lungs, blood, liver and kidneys were 3.5–5.3 mg/g, 4.1 mg/mL, 11.2-13.2 mg/g and 8.0–9.5 mg/g, respectively. DMS was observed in the tissue at about the same level. The DMDS concentration in the blood was 3.1 mg/mL.

However, in the current case, there was a two-month delay in the autopsy, resulting in putrefaction. The problem of autopsy delay occurs often in China. Normally, biosamples are collected within 48 hours after death, and the longest post-mortem interval is one week if the body is preserved by freezing. This case is a factory accident, and no autopsy would be done if corporate representatives of the factory and family members of the deceased come to an agreement. However, the family members did not accept compensation at that time. Therefore, the case was taken to court. The local court accepted this case, and the autopsy was entrusted to the Institute of Forensic Science, Ministry of Justice, PRC. No MT was detected in the biosamples. In addition, high concentrations of DMDS were found in the lungs and livers.

In the body, MT is partly methylated to DMS [8]. Additionally, MT is oxidized to DMDS and DMTS, and the formation of these compounds is related to the MT content [16]. A part of the detected DMS and DMDS could be from MT metabolization and oxidization. Terazawa et al. [20] reported a fatal case due to inhalation of DMS in a confined space. In the atmosphere of the confined space, MT was present at a concentration of less than 10×10^−6^, DMS was present at several ppm and DMDS was present at less than 1×10^−6^. The DMS concentration range was 0.01–0.23 mg/g in the tissue of the victim.

In mammalian tissues, there is a conversion between MT and H_2_S. The enzyme thiol S-methyltransferase (EC 2.1.1.9) can convert H_2_S into MT [6,21]. On the other hand, MT can be first demethylated to H_2_S, and H_2_S can be then oxidized to thiosulphate and sulphate [22]. Although hydrogen sulphide is the most toxic of these substances, considerable concentrations of organic reduced sulphur compounds, such as MT, DMS and DMDS, can be found, for example, in pulp mills [23]. In this case, the fatal intoxication by inhalation of VSCs was confirmed.

Sulphide levels were determined in all the blood, liver and lung samples from V#1 and V#2. The concentrations of sulphide in the blood samples were approximately 26 times higher than the level (below 0.05 μg/mL) in healthy persons [24] and were similar or higher than the levels reported in fatal cases in humans [25]. Considering the post-mortem production of sulphide due to putrefaction of the blood and tissues, thiosulphate should be analysed as an indicator of hydrogen sulphide poisoning [22].

Until now, there have been few reports on the concentration of DMDS in biosamples from post-mortem cases. The major reason for the lack of reports is that DMDS was not included as a target compound in previous analytical methods.

## Conclusion

Acute fatal poisoning due to the inhalation of toxic gas frequently occurs in China. HS-GC/FID with two columns of different polarities was utilized to screen MT, DMS, DMDS and DMTS in blood. The method developed in this report was applied to a fatal accident case. MT is easily oxidized. After about two months of the death, no MT was detected in the biosamples. DMS and DMDS were detected in the lungs at concentrations of 0.5 and 1.3 mg/g and 2.2 and 4.1 mg/g, respectively. DMDS liver concentrations were 2.5 and 6.5 mg/g. In addition to H_2_S, screening for additional volatile sulphur compounds could help establish the cause of death.

## References

[1] Li XL Implement of nosocomial rescue and emergency management measures for emergent group mixed gas poisoning. China Pract Med. 2016;7:260–261. Chinese.

[2] Song JB A hydrogen sulfide poisoning case happened in toilet. J Forensic Med. 2014;30:229–230. Chinese.

[3] Niu YM, Hao FT Study on prevention and treatment of acute irritating gas poisoning. Occup Health Emerg Rescue. 2012;30:190–193. Chinese.

[4] Lu Y, Li X, You Z Treatment effects of hyperbaric oxygen on 162 patients of acute harmful gas poison. Chongqing Med. 2008;37:2535–2536. Chinese.

[5] Kangas J, Jäppinen P, Savolainen H Exposure to hydrogen sulfide, mercaptans and sulfur dioxide in pulp industry. Am Ind Hyg Assoc J. 1985;45:787–790. 10.1080/15298668491400647 6517022

[6] Tangerman A Measurement and biological significance of the volatile sulfur compounds hydrogen sulfide, methanethiol and dimethyl sulfide in various biological matrices. J Chromatogr B. 2009;877:3366–3377. 10.1016/j.jchromb.2009.05.026 19505855

[7] Bosch PLF, Graaff M, Fortuny-Picornell M, et al. Inhibition of microbiological sulfide oxidation by methanethiol and dimethyl polysulfides at natron-alkaline conditions. Appl Microbiol Biotechnol. 2009;83:579–587. 19333598 10.1007/s00253-009-1951-6 PMC7419365

[8] Jäppinen P, Kangas J, Silakoski L, et al. Volatile metabolites in occupational exposure to organic sulfur compounds. Arch Toxikol. 1993;67:104–106. 10.1007/BF01973679 8481097

[9] Kage S, Nagata T, Kimura K, et al. Extractive alkylation and gas chromatographic analysis of sulfide. J Forensic Sci. 1988;33:217–222. 3351460

[10] Burbank HM, Qian MC Volatile sulfur compounds in Cheddar cheese determined by headspace solid-phase microextraction and gas chromatograph-pulsed flame photometric detection. J Chromatogr A. 2005;1066:149–157. 15794566 10.1016/j.chroma.2005.01.027

[11] López R, Lapeña AC, Cacho J, et al. Quantitative determination of wine highly volatile sulfur compounds by using automated headspace solid-phase microextraction and gas chromatography-pulsed flame photometric detection : Critical study and optimization of a new procedure. J Chromatogr A. 2007;1143:8–15. 17207804 10.1016/j.chroma.2006.12.053

[12] Zhang M, Chen L Continuous underway measurements of dimethyl sulfide in seawater by purge and trap gas chromatography coupled with pulsed flame photometric detection. Mar Chem. 2015;174:67–72.

[13] Kinsela AS, Reynolds JK, Melville MD Agricultural acid sulfate soils: a potential source of volatile sulfur compounds?. Environ Chem. 2007;4:18–25.

[14] Peters F, Drummer O, Musshoff F Validation of new methods. Forensic Sci Int. 2007;165:216–224. 16781833 10.1016/j.forsciint.2006.05.021

[15] Qiang HS, Chen H, Shen BH, et al. Determination of sulfide in blood from hydrogen sulfide poisoning cases. Fa Yi Xue Za Zhi. 2017;33:148–153. Chinese. 29231020 10.3969/j.issn.1004-5619.2017.02.008

[16] Martínez-Cuesta MDC, Requena CPT Methionine metabolism: major pathways and enzymes involved and strategies for control and diversification of volatile sulfur compounds in cheese. Crit Rev Food Sci Nutrition. 2013;53:366–385. 23320908 10.1080/10408398.2010.536918

[17] And MCI, Larachi F Solubility of total reduced sulfurs (hydrogen sulfide, methyl mercaptan, dimethyl sulfide, and dimethyl disulfide) in liquids. J Chem Eng Data. 2006;52:2–19.

[18] Giri BS, Kim KH, Pandey RA, et al. Review of biotreatment techniques for volatile sulfur compounds with an emphasis on dimethyl sulfide. Process Biochem. 2014;49:1543–1554.

[19] Takahama K, Kikuda Y A fatal case of acute methylmercaptan poisoning.Res Pract Forensic. 1986;29:101–106. Japanese.

[20] Terazawa K, Mizukami K, Wu B, et al. Fatality due to inhalation of dimethyl sulfide in a confined space: a case report and animal experiments. International Journal of Legal Medicine. 1991;104:141–144. 1911412 10.1007/BF01369718

[21] Lomans BP, Drift CVD, Pol A, et al. Microbial cycling of volatile organic sulfur compounds. Cell Mol Life Sci. 2001;59:3341–3352. 10.1007/s00018-002-8450-6 PMC11337448 12022467

[22] Kage S, Takekawa K, Kurosaki K, et al. The usefulness of thiosulfate as an indicator of hydrogen sulfide poisoning: three cases. Int J Leg Med. 1997;110:220–222. 10.1007/s004140050071 9274948

[23] Smet E, Langenhove HV Abatement of volatile organic sulfur compounds in odorous emissions from the bio-industry. J Chromatogr. 1998;881:569–581. 10.1023/a:1008281609966 10022070

[24] McAnalley BH, Lowry WT, Oliver RD, et al. Determination of inorganic sulfide and cyanide in blood using specific ion electrodes: application to the investigation of hydrogen sulfide and cyanide poisoning. J Anal Toxicol. 1979;3:111–114.

[25] Maebashi K, Iwadate K, Sakai K, et al. Toxicological analysis of 17 autopsy cases of hydrogen sulfide poisoning resulting from the inhalation of intentionally generated hydrogen sulfide gas. Forensic Sci Int. 2011;207:91–95. 20965672 10.1016/j.forsciint.2010.09.008

